# Decision curve analysis to identify optimal candidates of liver resection for intermediate-stage hepatocellular carcinoma with hepatitis B cirrhosis: A cohort study

**DOI:** 10.1097/MD.0000000000031325

**Published:** 2022-10-28

**Authors:** He Li, Siyu Chen, Linbin Lu, Xinyu Hu, Shan Lin, Lijun Zhu

**Affiliations:** a Department of General Surgery, the 900th Hospital of Joint Logistic Support Force, PLA, Fuzong Clinical College of Fujian Medical University, Fuzhou, Fujian, PR China; b Department of Oncology, the 900th Hospital of Joint Logistic Support Force, PLA, Fuzong Clinical College of Fujian Medical University, Fuzhou, Fujian, PR China; c Department of Neurology, the 900th Hospital of Joint Logistic Support Force, PLA, Fuzong Clinical College of Fujian Medical University, Fuzhou, Fujian, PR China.

**Keywords:** decision curve analysis, hepatic resection, hepatocellular carcinoma, net benefit, transarterial chemoembolization

## Abstract

The selection criterion for liver resection (LR) in intermediate-stage (IM) hepatocellular carcinoma (HCC) is still controversial. This study aims to compare LR and transarterial chemoembolization (TACE) in the range of predicted death risk The multivariable Cox regression model (MVR) was estimated to predict mortality at 5 year. The cutoff values were determined by a 2-piece-wise linear regression model, decision curve analysis with MVR model, and hazard ratio curve for treatment plotted against the predicted mortality. 825 IM-hepatocellular carcinoma (IM-HCC) with hepatitis B cirrhosis were included for analysis (TACE, *n* = 622; LR, *n* = 203). The 5-year overall survival (OS) rate of LR patients was higher than the TACE group (52.8% vs 20.8%; *P* < .0001). The line of LR and TACE were crossing with predicted death risk at 100% (*P* for interaction = .008). The benefit of LR versus TACE decreased progressively as predicted death risk > 0.55 (95%CI: 0.45, 0.62). When predicted death risk over 0.7, decision curve analysis suggested that LR and TACE did not increase net benefit. Patients were then divided into 4 subgroups by the cutoff values (<0.45, 0.45≥/<0.62, 0.62≥/<0.7, ≥0.7). The stratified analysis of treatment in different subgroups, hazard ratios were 0.39 (95%CI: 0.27, 0.56), 0.36 (95%CI: 0.23, 0.56), 0.51 (95%CI: 0.27, 0.98), and 0.46 (95%CI: 0.27, 0.80), respectively. LR reached the maximal relative utility in the interval of 0.45 to 0.62, and both LR and TACE did not increase net benefit at the 5-year death risk over 0.7.

## 1. Introduction

Hepatocellular carcinoma (HCC) is the fifth cause of death in China, and its high levels of morbidity and mortality rates mostly result from hepatitis B cirrhosis, with nearly half of the global new cases and deaths.^[1,2]^ Liver resection (LR), regarded as a curative therapy, is widely performed for the patients with Barcelona Clinic Liver Cancer (BCLC) 0-A stage HCC in China, even for the resectable intermediate-stage (IM) tumor.^[3]^ According to the last BCLC staging system, transarterial chemoembolization (TACE) has been recommended as the first-line treatment for unresectable IM-HCC.^[4]^ In the clinical setting, however, TACE is also applied on a wide scale in resectable HCC patients alone or combined with surgery. The advantage of surgery over TACE has already been extensively described in the resectable BCLC-B HCC patients^[5]^ and the cirrhotic patients.^[6]^ However, which groups are more suitable for LR remains an open issue.

Recently, an NSP scoring system (1, >1 point; median OS, 61.3 vs 19.3 months) was developed to select patients with IM-HCC for LR accurately.^[7]^ Compared with the TACE treatment, surgical resection has the better survival only in the subclass of BCLC-B HCC with a Child-Pugh score of 5 and no more than 3 tumors.^[8]^ Besides, according to the modified Bolondi’s sub-staging model, partial hepatectomy has an optimal long-term survival than TACE in the BCLC-B1/B2 HCC.^[9]^ Interestingly, in another study, patients were grouped into low-risk (3 year mortality ≤ 35%), medium-risk (35% < 3 year mortality < 70%), and high-risk (3 year mortality ≥ 70%) groups by regret-based decision curve analysis. And only the patients in the low-risk group have a better outcome than those treated with TACE.^[10]^ However, more convenient biomarkers are urgent to choose suitable therapy for IM-HCC, a highly heterogeneous population. In the present study, we use a predicted mortality risk-based decision analysis to compare the clinical outcome of hepatectomy and TACE in the IM-HCC. We present the following article in accordance with the STROBE reporting checklist.

## 2. Material and Methods

### 2.1. Patient selection

Clinical and biological data have been previously published in full,^[11,12]^ and the details of inclusion criteria, diagnosis, and treatment are shown in Figure S1, http://links.lww.com/MD/H754, Supplemental Content, which illustrates inclusion and exclusion criteria of HCC patients.

The Ethics Committee approved the study protocol (2017-FXY-129) of Sun Yat-sen University Cancer Center and another 3 medical centers.^[11]^ Because this was a retrospective study, the informed consent was waived.

### 2.2. Definition and follow up

LR: surgical therapy for hepatic segments or lobes lesions. In the clinical, patients with well liver function and less tumor loading were usually suitable for LR. The optimal treatment for each HCC patient was based on the decisions of the multidisciplinary teams.^[12]^ The indications for LR in HCC patients were appropriate residual liver volume determined by computed tomography. Thirty percent remnant liver volume after LR was considered adequate for patients without cirrhosis. And it should be more than 50% for those with chronic hepatitis, cirrhosis, and severe fatty liver. Besides, LR was a contraindication among the patients with intermediate, or advanced cirrhosis, or poor liver function (Child–Pugh C). Patients who satisfied the indications for LR were treated by surgical resection, unless requested TACE.

TACE: conventional chemoembolization of the hepatic artery. After embolization, angiography was performed to determine the extent of vascular occlusion and to assess blood flow in other arterial vessels. Lesions exceeding 1 cm in diameter were embolized superselectively, and less than 1 cm for non-selectively. According to the response to the TACE, the subsequent therapies include ablative therapies, surgical resection, TACE, or targeted therapies.

Overall survival (OS): the time between the beginning of LR/TACE and the last following-up or death for any cause.

During the initial treatment period for the first 2 years, patients were followed up for every 2 or 3 months if complete remission was achieved. The frequency gradually decreased to every 3 to 6 months after remission of 2 years.

### 2.3. Statistical analyses

To compare differences of baseline characteristics between the LR and TACE groups, we compared categorical variables with the chi-square test and continuous variables by the Mann–Whitney test. Kaplan–Meier methods calculated OS rates for the LR and TACE cohorts. Statistical analysis was performed using Empower (www.empowerstats.com, X&Y solutions, inc. Boston MA) and R software (version 3.4.3). *P* value < .05 considered significant.

To explore the optimal range of death risk for the net benefit of LR against TACE, we divide the statistical analyses into 3 steps:

Firstly, according to the previously reported methods,^[13]^ we developed the multivariable Cox regression (MVR) model, which was based on the significantly different covariates between 2 treatment groups (all *P* < .05 list in Table 1), including the continuous covariates of age, PT and diameter of the main tumor, as well as the categorical covariates *No*. of intrahepatic lesions, both lobe with lesions.

**Table 1:** Baseline characteristics between TACE and surgery group in the derivation cohort.

	Treatment		Standardize difference	*P* value
	TACE	Surgery		
No.	622	203		
Age	53.9 ± 12.2	50.6 ± 12.4	0.3 (0.1, 0.4)	.001
Gender			0.0 (−0.1, 0.2)	.716
Male	566 (91.0%)	183 (90.1%)		
Female	56 (9.0%)	20 (9.9%)		
Log AFP (ng/mL)	2.6 ± 1.4	2.4 ± 1.5	0.2 (−0.0, 0.3)	.050
<200	271 (45.5%)	98 (51.0%)	0.1 (−0.1, 0.3)	.185
≥200	324 (54.5%)	94 (49.0%)		
PT (sec)	12.3 ± 1.3	12.5 ± 1.6	0.2 (0.0, 0.3)	.037
ALB (g/L)	39.0 ± 5.8	38.4 ± 5.4	0.1 (−0.1, 0.3)	.220
Log TBLT (umol/L)	1.3 ± 0.2	1.3 ± 0.3	0.1 (−0.0, 0.3)	.157
Major tumor size (mm)	75.2 ± 37.0	63.6 ± 27.6	0.4 (0.2, 0.5)	<.001
No. of intrahepatic lesions			0.7 (0.5, 0.9)	<.001
2	150 (24.1%)	110 (54.2%)		
3	51 (8.2%)	20 (9.9%)		
>3	421 (67.7%)	73 (36.0%)		
Location of lesions			0.6 (0.5, 0.8)	<.001
Left/right	219 (35.2%)	132 (65.0%)		
Both	403 (64.8%)	71 (35.0%)		
Child-Pugh class			0.1 (−0.1, 0.3)	.246
A	522 (86.9%)	168 (83.6%)		
B	79 (13.1%)	33 (16.4%)		

Numbers that do not add up to 825 are attributable to missing data. The chi-square test was performed for categorical measures and Kruskal–Wallis test for continuous measures.

AFP = alpha-fetoprotein, ALB = albumin, PT = prothrombin time, TACE = transarterial chemoembolization, TBLT = total bilirubin.

To evaluate the interaction between LR and death risk, we used the method^[13]^: predicted OS rate at 5 year (Pi) was calculated based on the MVR model in the LR group. From this model, death risk at 5 year (1-Pi) was also the baseline death risk for both LR and TACE cohorts. This predicted death risk (1-Pi) was added as a covariate to the second multivariate Cox model to calculate the predicted probability of survival at 5 year (Pii), and spline smoothing curve between 5 year predicted death risk (1-Pi) and probability (Pii) were graphed stratified by treatment through the generalized additive model. Furthermore, a 2-piece-wise linear regression and the recursive method were performed to calculate the inflexion point of the TACE line, and a log-likelihood ratio test was used to compare the 1-line linear regression. The 95% confidence interval of inflection point was confirmed by 500 bootstrap resampling, which may be the optimal range for the net benefit of LR against TACE. We used inverse probability of treatment weighting (IPTW) to eliminate the differences between 2 groups, and repeat the first step.

Secondly, to further verify this interval, we used the “ggDCA” R package to establish a decision curve analysis with the MVR model. We calculated the net benefit of the model and determined the cutoff value through 2 reference strategies (test none or test all).

Thirdly, to further validate the cutoff points from the 2 steps above, we paint the hazard ratio (HR) curve (LR vs TACE) for the 5 year predicted death risk by the Cox regression model. The stratified analyses were performed to explore LR (vs TACE) hazard ratio in each subgroup based on the observed cutoff values.

## 3. Results

### 3.1. Descriptive characteristics

In the derivation cohort, 825 patients with hepatitis B cirrhosis were included in the final analysis, with TACE (n = 622, 75.4%) or surgical resection (n = 203, 24.6%) as their first-line treatment. Table 1 showed the baseline features of study populations. The univariable analysis results were listed in Table S1, http://links.lww.com/MD/H752, Supplemental Content, which focused on the markedly different covariates between the 2 groups.

The median OS for the entire cohort was 23.3 (95%CI: 20.0, 26.6) month. And it was 18.0 (95%: 16.2, 19.9) month for TACE group versus 67.4 (95%CI: 44.0, NA) month for LR group (*P* < .0001, see Fig. S2, http://links.lww.com/MD/H755, Supplemental Content, which showed Kaplan–Meier curves of OS in the derivation cohort stratified with LR and TACE). The OS rates at 5 year was 29.5% (95%CI: 25.7%, 33.9%) for the entire cohort, and it was 52.8% (95% CI: 45.0%, 62.0%) for LR group versus 20.8% (95%CI: 16.8%, 25.7%) for the TACE group (*P* < .0001).

### 3.2. Survival analysis for net benefit in treatment cohorts

**Figure 1.**
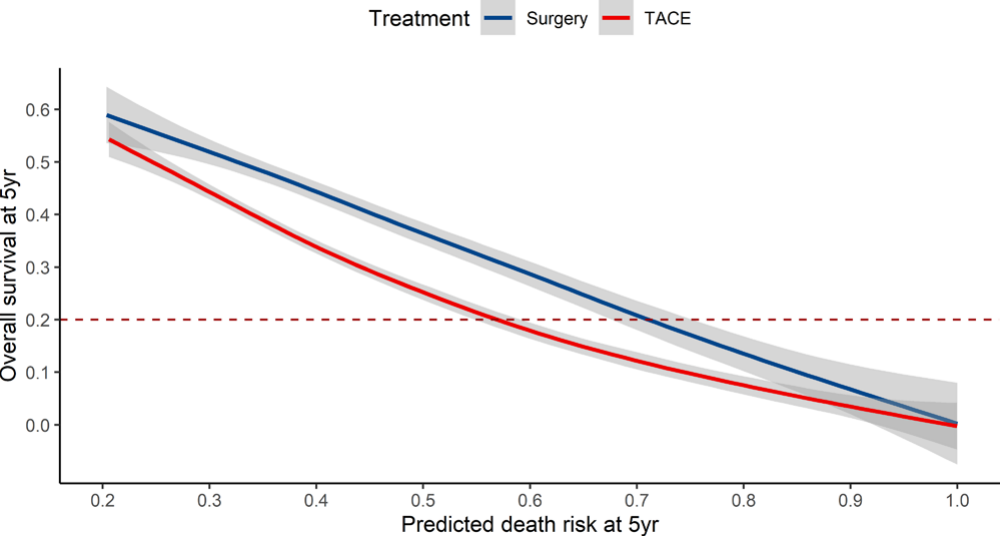
Overall survival rate plotted against predicted probability of death at 5 yr. The blue line indicates hepatic resection for primary treatment. The red line indicates TACE for primary treatment. Dashed line indicates observed OS at 5 yr for the TACE group (20.8%, 95%CI: 16.8%, 25.7%). The inflexion point of the TACE line was calculated at 0.55 (95%CI: 0.45, 0.62). OS = overall survival, TACE = transarterial chemoembolization.

The MVR model was estimated by age, PT, largest tumor size, No. of intrahepatic lesions, and both lobes with lesions, which was used to predict 5-year death risk and OS rate for the entire cohort (see Fig. S3, http://links.lww.com/MD/H756, Supplemental Content, which illustrates nomogram to predict the OS). Its Harrell’s C index was 0.70 (0.67, 0.72). In Figure 1, the predicted death risk was plotted against OS rate at 5 year. And the lines for LR and TACE crossed at 1.0 (*P* for interaction = .008). In Table 2, we found that the inflexion point of the TACE line was calculated at 0.55 (95%CI: 0.45, 0.62), indicating the benefit from LR decreased progressively as predicted OM risk over 55%. Interestingly, the predicted survival rate of the TACE line at 0.55 was 0.20 (95%CI: 0.18, 0.21), which was in the interval of observed OS at 5 year for the TACE group (20.8%, 95%CI: 16.8%, 25.7%). The results of IPTW were consistent and robust (see Fig. S4/5, http://links.lww.com/MD/H757, Supplemental Content, which shows standardized differences of the 5 covariates after propensity-matching, and OM -free survival rate plotted against predicted probability of OM at 5 year after IPTW; see Table S2, http://links.lww.com/MD/H753, Supplemental Content, which shows threshold effect analysis of TACE group in the derivation cohort using 2-piece-wise linear regression after IPTW).

**Table 2:** Threshold effect analysis of TACE group in the derivation cohort using 2-piece-wise linear regression.

	Unadjusted β(SD)	*P* value
The 1-line linear model	−0.71 (0.02)	<.0001
The 2-piece-wise linear model		
<0.55	−0.98 (0.05)	<.0001
>0.55	−0.47 (0.04)	<.0001
*P* forlog-likelihood ratio test		<.001

The predicted value at the point of 0.55 (95%CI: 0.45, 0.62) was 0.20 (95%CI: 0.18, 0.21). A log-likelihood ratio test was used to compare the 1-line linear regression.

TACE = transarterial chemoembolization.

**Figure 2.**
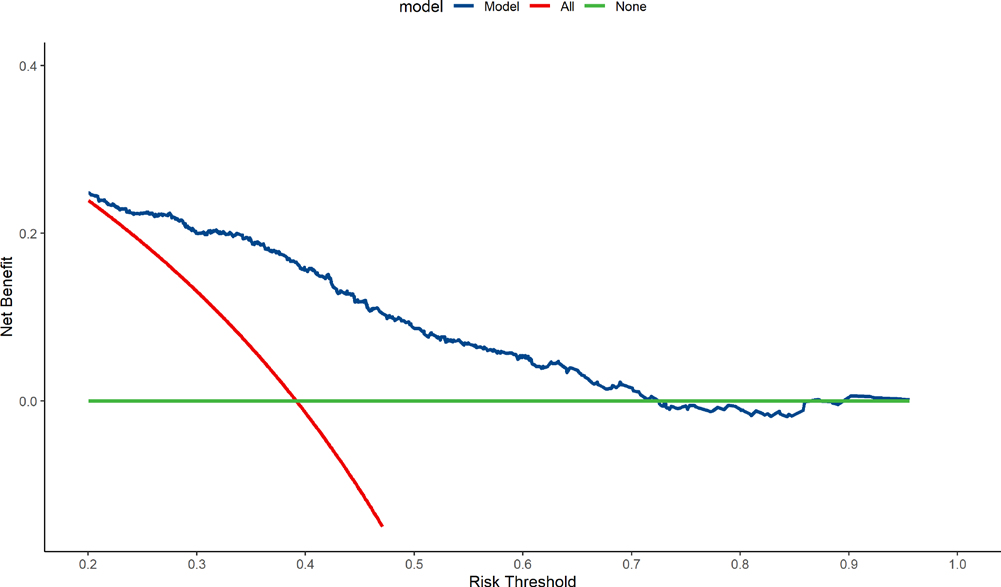
Decision curve analysis with multivariable Cox regression model for overall survival. Solid green line = net benefit when all BCLC-B HCC is considered not having the outcome; red dashed line = net benefit when all pregnant women are considered the outcome. BCLC = Barcelona clinic liver cancer, HCC = hepatocellular carcinoma.

**Figure 3.**
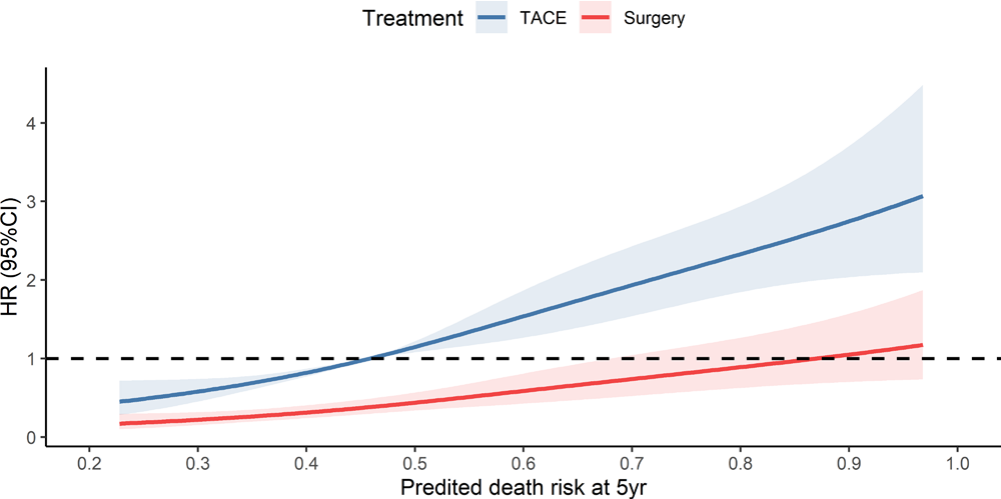
Hazard ratio of treatment plotted against the predicted mortality at 5 yr. Hazard ratios for LR versus TACE in the stratified analysis are 0.39 (95%CI: 0.27, 0.56), 0.36 (95%CI: 0.23, 0.56), 0.51 (95%CI: 0.27, 0.98), and 0.46 (95%CI: 0.27, 0.80) for each subclass (<0.45, 0.45≥/<0.62, 0.62≥/<0.7, ≥0.7). LR = liver resection, TACE = transarterial chemoembolization.

As was shown in Figure 2, net benefit curves suggested none could receive a net benefit from surgery and TACE for death risk > 0.7; maximal relative utility occurred at 0.4. Consistently, these cutoff values were further validated by the hazard ratio curve of LR and TACE against the 5 year predicted death risk, which was shown in Figure 3. Thus, patients were grouped into 4 subclasses: R5 < 0.45, 0.45 ≥ R5 < 0.62, 0.62 ≥ R5 < 0.7, R5 ≥ 0.7. Figure 3 also illustrated the stratified analysis of LR (vs TACE), hazard ratios were 0.39 (95%CI: 0.27, 0.56), 0.36 (95%CI: 0.23, 0.56), 0.51 (95%CI: 0.27, 0.98), and 0.46 (95%CI: 0.27, 0.80) for each subclass.

## 4. Discussion

In this large-scale, real-world data, we found that the OS for LR was significantly better than their TACE counterparts, which was consistent with the previous literature.^[5,14]^ However, the net benefit from LR decreased progressively as predicted death risk > 55%, with the interval of maximal relative utility ranging from 45% to 62%. When death risk > 70%, LR and TACE did not increase net benefit.

In 2015, Colombo et al^[15]^ had come up with an assumption that IM-HCC patients could still be suitable for LR if the 5-year survival rate reached 50%. Our findings primarily validated this hypothesis. In line with the previous literature,^[9,16]^ we identified a subgroup with death risk < 70%, in which patients treated with LR had significantly better OS than the TACE group. Based on Bolondi’s sub-staging model,^[17]^ Wei et al,^[9]^ patients’ postoperative 5-year survival rate in the BCLC B1-B3 stage was 49.5%, 33.7%, and 12.9%, respectively. Nevertheless, only the BCLC B1/B2 had more optimal long-term survival than the TACE group. In another large-scale study,^[16]^ the benefit from LR was observed in the patients of BCLC-B1/B2 but not B3/B4.

Our study had some strengths. We used 3 novel methods to calculate the cutoff accuracy value to evaluate the tumor loading. Besides, in our study, the survival rates between LR and TACE were compared in the vast and continuous range so that the exact cutoff values could be calculated. When evaluating the role of LR among patients with anatomically resectable tumors and well liver function, the randomized control trial was obviously against medical ethics. Therefore, real-world data’s predicted mortality risk-based decision analysis may be better.

Our study also had several limitations. Firstly, this was a secondary analysis based on open-access data. The surgical program (radical or palliative; segmentectomy, lobectomy, or non-anatomical) was unclear. The details of cirrhosis rates and portal hypertension were also unknown. Besides, residual bias and unmeasured confounders were unavoidable. Secondly, the percentage of resectable HCC patients in the TACE group with 5-year death risk < 70% was unclear. However, it was worthy of note that the potential unresectable HCC patients treated with TACE resulting from such errors would bias toward the null and lead to an underestimation of the net benefit from LR versus TACE.

## 5. Conclusions

LR reached the maximal relative utility in the interval of 0.45 to 0.62, and both LR and TACE did not increase net benefit at the 5-year death risk over 0.7.

## Author contributions

**Data curation:** Linbin Lu.

**Funding acquisition:** Lijun Zhu.

**Investigation:** He Li.

**Methodology:** Linbin Lu.

**Project administration:** He Li.

**Resources:** Xinyu Hu.

**Software:** Siyu Chen, Shan Lin.

**Supervision:** Lijun Zhu.

**Writing – original draft:** He Li, Siyu Chen, Linbin Lu, Xinyu Hu, Shan Lin, Lijun Zhu.

**Writing – review & editing:** He Li, Siyu Chen, Linbin Lu, Xinyu Hu, Shan Lin, Lijun Zhu.

## Acknowledgments

We gratefully the statistical support from Empower U team of the Department of Epidemiology and Biostatistics, X&Y solutions Inc.

## Supplementary Material



## References

[1] Zhou M Wang H Zeng X. Mortality, morbidity, and risk factors in China and its provinces, 1990–2017: a systematic analysis for the global burden of disease study 2017. Lancet (London, England). 2019;394:1145–58. 10.1016/S0140-6736(19)30427-1 PMC6891889 31248666

[2] Bray F Ferlay J Soerjomataram I. Global cancer statistics 2018: GLOBOCAN estimates of incidence and mortality worldwide for 36 cancers in 185 countries. CA Cancer J Clin. 2018;68:394–424. 30207593 10.3322/caac.21492

[3] Zhou J Sun HC Wang Z. Guidelines for diagnosis and treatment of primary liver cancer in China (2017 Edition). Liver Cancer. 2018;7:235–60. 30319983 10.1159/000488035 PMC6167671

[4] Liver EAftSot. EASL clinical practice guidelines: management of hepatocellular carcinoma. J Hepatol. 2018;69:182–236. 29628281 10.1016/j.jhep.2018.03.019

[5] Hyun MH Lee YS Kim JH. Hepatic resection compared to chemoembolization in intermediate- to advanced-stage hepatocellular carcinoma: a meta-analysis of high-quality studies. Hepatology. 2018;68:977–93. 29543988 10.1002/hep.29883

[6] Chen S Jin H Dai Z. Liver resection versus transarterial chemoembolization for the treatment of intermediate-stage hepatocellular carcinoma. Cancer Med. 2019;8:1530–9. 30864247 10.1002/cam4.2038 PMC6488138

[7] Zhang YF Zhou J Wei W. Intermediate-stage hepatocellular carcinoma treated with hepatic resection: the NSP score as an aid to decision-making. Br J Cancer. 2016;115:1039–47. 27701389 10.1038/bjc.2016.301 PMC5117793

[8] Tada T Kumada T Toyoda H. Role of hepatic resection in patients with intermediate-stage hepatocellular carcinoma: a multicenter study from Japan. Cancer Sci. 2017;108:1414–20. 28406546 10.1111/cas.13257 PMC5497930

[9] Wei WX Yang ZS Lu LH. Long-term survival after partial hepatectomy for sub-stage patients with intermediate stage hepatocellular carcinoma. Int J Surg (London, England). 2018;56:256–63. 10.1016/j.ijsu.2018.06.020 29935368

[10] Cucchetti A Djulbegovic B Tsalatsanis A. When to perform hepatic resection for intermediate-stage hepatocellular carcinoma. Hepatology. 2015;61:905–14. 25048515 10.1002/hep.27321

[11] Shen L Zeng Q Guo P. Dynamically prognosticating patients with hepatocellular carcinoma through survival paths mapping based on time-series data. Nat Commun. 2018;9:2230. 29884785 10.1038/s41467-018-04633-7 PMC5993743

[12] Lu L Zheng P Wu Z. Hepatic resection versus transarterial chemoembolization for intermediate-stage hepatocellular carcinoma: a cohort study. Front Oncol. 2021;11:618937. 34778022 10.3389/fonc.2021.618937 PMC8579001

[13] Loppenberg B Dalela D Karabon P. The impact of local treatment on overall survival in patients with metastatic prostate cancer on diagnosis: a national cancer data base analysis. Eur Urol. 2017;72:14–9. 27174537 10.1016/j.eururo.2016.04.031

[14] Liang L Xing H Zhang H. Surgical resection versus transarterial chemoembolization for BCLC intermediate stage hepatocellular carcinoma: a systematic review and meta-analysis. HPB (Oxford). 2018;20:110–19. 29174493 10.1016/j.hpb.2017.10.004

[15] Colombo M Sangiovanni A. Treatment of hepatocellular carcinoma: beyond international guidelines. Liver Int. 2015;35(Suppl 1):129–38. 25529098 10.1111/liv.12713

[16] Kariyama K Nouso K Wakuta A. Treatment of intermediate-stage hepatocellular carcinoma in Japan: position of curative therapies. Liver Cancer. 2020;9:41–9. 32071908 10.1159/000502479 PMC7024876

[17] Bolondi L Burroughs A Dufour JF. Heterogeneity of patients with intermediate (BCLC B) hepatocellular carcinoma: proposal for a subclassification to facilitate treatment decisions. Semin Liver Dis. 2012;32:348–59. 23397536 10.1055/s-0032-1329906

