# Supplementary material for: Decision curve analysis to identify optimal candidates of liver resection for intermediate-stage hepatocellular carcinoma with hepatitis B cirrhosis: A cohort study
Source: Medicine (Baltimore). 2022 Oct 28;101(43):e31325. doi: 10.1097/MD.0000000000031325 (PMC9622667; doi:10.1097/MD.0000000000031325)
Supplement: Supplementary file 1 [file medi-101-e31325-s001.pdf]

Table S1. The univariate analysis focusing on the derivation cohort

	Statistics	Death	P-value
Treatment			
TACE	622 (75.4%)	Reference	
LR	203 (24.6%)	0.39 (0.30, 0.50)	<0.0001
Age	53.2 ± 12.5	1.00 (0.99, 1.01)	0.661
Gender			0.381
male	749 (90.8%)	Reference	
female	76 (9.2%)	1.15 (0.84, 1.58)	
ALB (g/L)	38.8± 5.7	0.99 (0.97, 1.00)	0.104
PT (second)	12.3 ± 1.4	1.09 (1.01, 1.16)	0.017
No. of intrahepatic lesions			
2	260 (31.5%)	Reference	
3	71 (8.6%)	1.11 (0.78, 1.59)	0.554
>3	494 (59.9%)	1.57 (1.28, 1.94)	<0.0001
Major tumor size(mm)	72.3 ± 35.3	1.01 (1.01, 1.02)	<0.0001
Both lobes with lesion			
no	351 (42.5%)	Reference	
yes	474 (57.5%)	1.44 (1.19, 1.74)	0.0001
$\log_{10}$ AFP (ng/ml)	2.5 ± 1.4	1.21 (1.13, 1.29)	<0.0001
$\log_{10}$ TBLT(umol/L)	1.3 ± 0.3	0.98 (0.67, 1.43)	0.909
Child-Pugh class			0.009
A	690 (86.0%)	Reference	
B	112 (14.0%)	1.41 (1.09, 1.83)	

Numbers that do not add up to 825 are attributable to missing data. AFP=alpha-fetoprotein, PT=prothrombin time, TBLT=total bilirubin, ALB=albumin, HR=hepatic resection.
