# Supplementary material for: Decision curve analysis to identify optimal candidates of liver resection for intermediate-stage hepatocellular carcinoma with hepatitis B cirrhosis: A cohort study
Source: Medicine (Baltimore). 2022 Oct 28;101(43):e31325. doi: 10.1097/MD.0000000000031325 (PMC9622667; doi:10.1097/MD.0000000000031325)
Supplement: Supplementary file 2 [file medi-101-e31325-s002.pdf]

Table S2. Threshold effect analysis of TACE group in the derivation cohort using two-piece-wise linear regression after IPTW.

	Adjusted β (95%CI)	P-value
The one-line linear model	-0.73 (-0.77, -0.69)	<0.0001
The two-piece-wise linear model		
< 56%	-0.92 (-1.01, -0.83)	<0.0001
≥ 56%	-0.54 (-0.63, -0.45)	<0.0001
P for log-likelihood ratio test		<0.001

The predicted value at the point of 56% was 0.23 (0.21, 0.24). A log-likelihood ratio test was used to compare the one-line linear regression
