# Supplementary figures and images for: Decision curve analysis to identify optimal candidates of liver resection for intermediate-stage hepatocellular carcinoma with hepatitis B cirrhosis: A cohort study
Source: Medicine (Baltimore). 2022 Oct 28;101(43):e31325. doi: 10.1097/MD.0000000000031325 (PMC9622667; doi:10.1097/MD.0000000000031325)

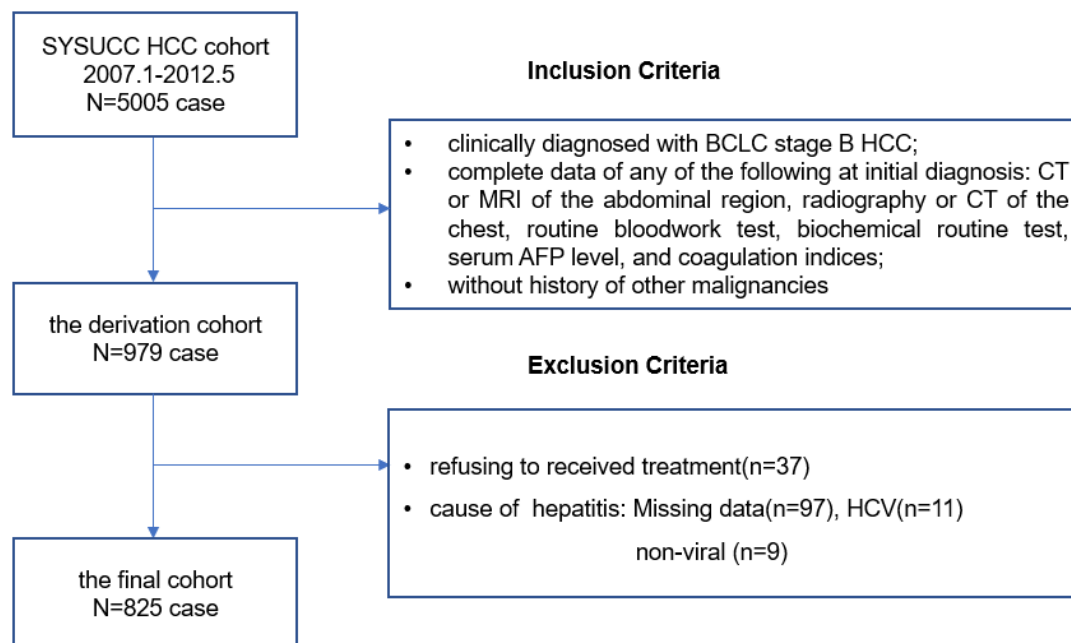

**Figure S1. Inclusion and exclusion criteria of hepatocellular carcinoma patients.**

Supplement: Supplementary file 3 [file medi-101-e31325-s003.pdf]

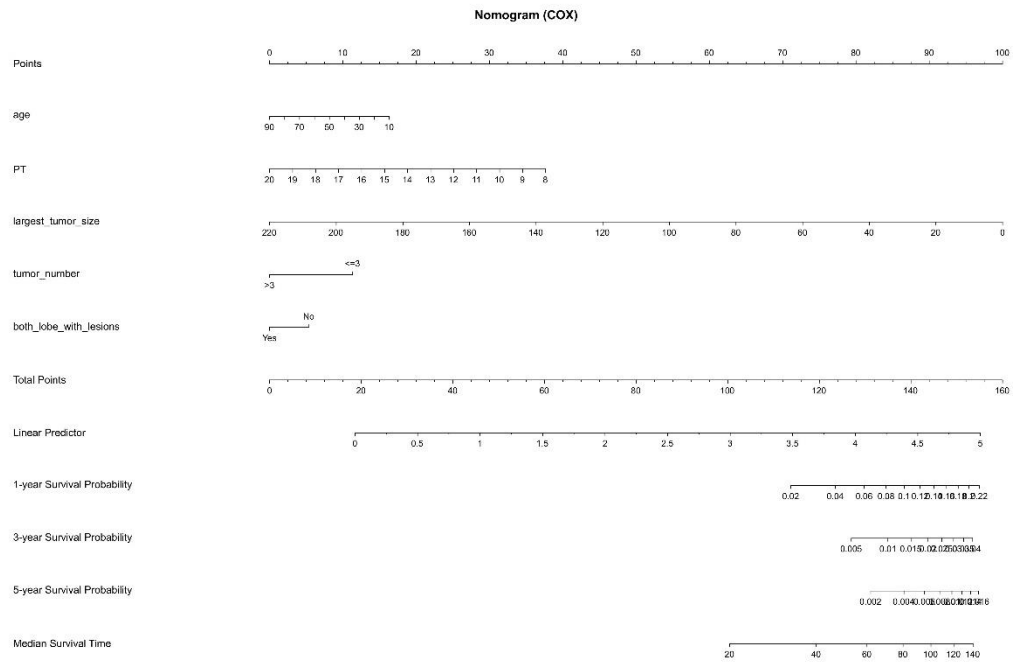

**Figure S3. Nomogram to predict the OS. 1yr-,3yr- and 5yr- AUC are 0.73, 0.69, 0.66.**

Supplement: Supplementary file 5 [file medi-101-e31325-s005.pdf]
