# Supplementary material for: Decision curve analysis to identify optimal candidates of liver resection for intermediate-stage hepatocellular carcinoma with hepatitis B cirrhosis: A cohort study
Source: Medicine (Baltimore). 2022 Oct 28;101(43):e31325. doi: 10.1097/MD.0000000000031325 (PMC9622667; doi:10.1097/MD.0000000000031325)
Supplement: Supplementary file 4 [file medi-101-e31325-s004.pdf]

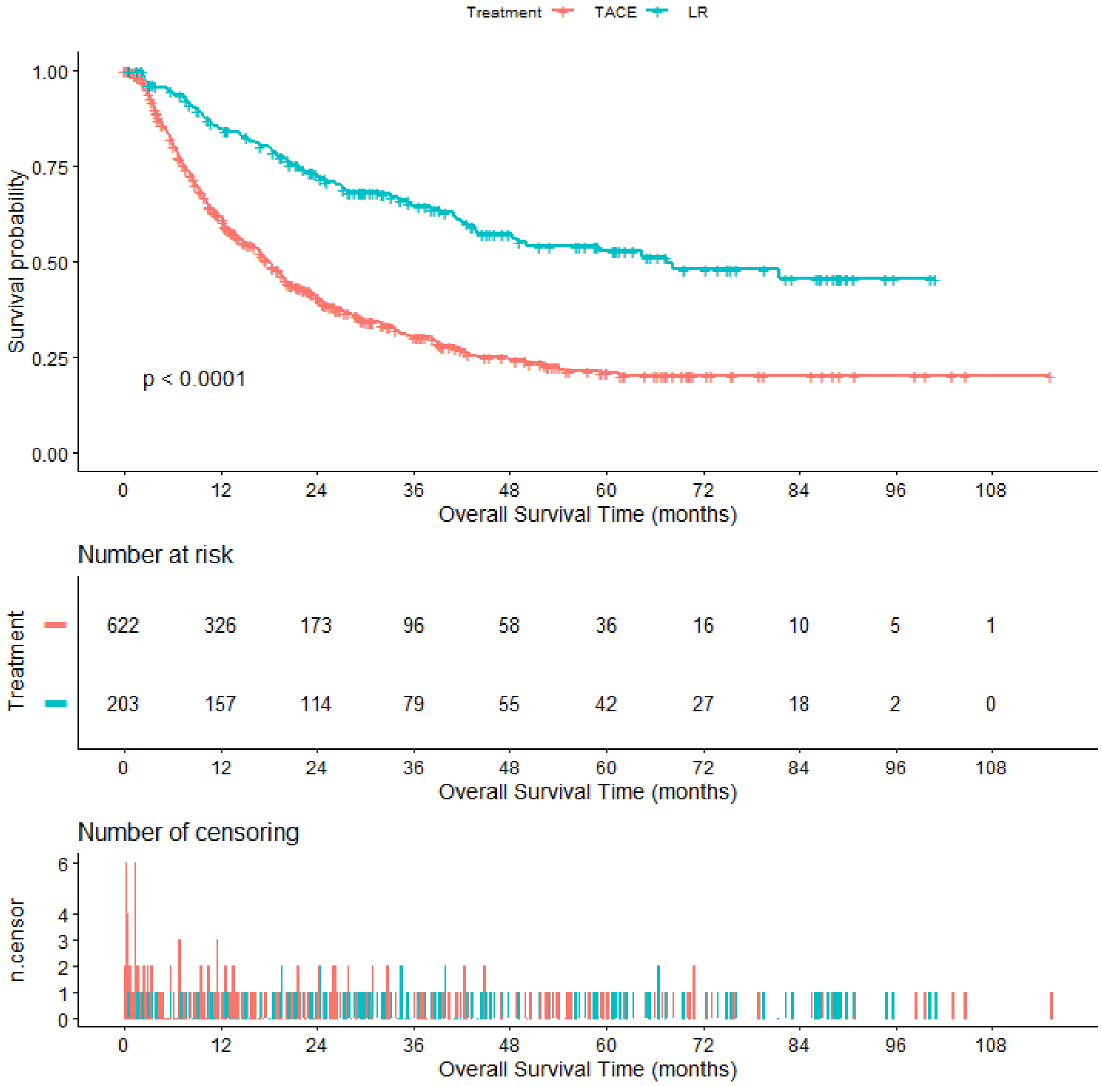

Figure S2. Kaplan-Meier curves of overall survival in the derivation cohort stratified with liver resection and transarterial chemoembolization.
